# Cure of Hookworm Infection with a Cysteine Protease Inhibitor

**DOI:** 10.1371/journal.pntd.0001680

**Published:** 2012-07-03

**Authors:** Jon J. Vermeire, Lorine D. Lantz, Conor R. Caffrey

**Affiliations:** 1 Department of Pediatrics, Section of Infectious Diseases, Yale University School of Medicine, New Haven, Connecticut; 2 Sandler Center for Drug Discovery, California Institute for Quantitative Biosciences and the Department of Pathology, University of California, San Francisco, San Francisco, California; Swiss Tropical and Public Health Institute, Switzerland

## Abstract

**Background:**

Hookworm disease is a major global health problem and principal among a number of soil-transmitted helminthiases (STHs) for the chronic disability inflicted that impacts both personal and societal productivity. Mass drug administration most often employs single-dose therapy with just two drugs of the same chemical class to which resistance is a growing concern. New chemical entities with the appropriate single-dose efficacy are needed.

**Methods and Findings:**

Using various life-cycle stages of the hookworm *Ancylostoma ceylanicum in vitro* and a hamster model of infection, we report the potent, dose-dependent cidal activities of the peptidyl cysteine protease inhibitors (CPIs) K11002 (4-mopholino-carbonyl-phenylalanyl-homophenylalanyl- vinyl sulfone phenyl) and K11777 (*N*-methylpiperazine-phenylalanyl-homophenylalanyl-vinylsulfone phenyl). The latter is in late pre-clinical testing for submission as an Investigational New Drug (IND) with the US Federal Drug Administration as an anti-chagasic. *In vitro*, K11002 killed hookworm eggs but was without activity against first-stage larvae. The reverse was true for K11777 with a larvicidal potency equal to that of the current anti-hookworm drug, albendazole (ABZ). Both CPIs produced morbidity in *ex vivo* adult hookworms with the activity of K11777 again being at least the equivalent of ABZ. Combinations of either CPI with ABZ enhanced morbidity compared to single compounds. Strikingly, oral treatment of infected hamsters with 100 mg/kg K11777 *b.i.d.* (i.e., a total daily dose of 200 mg/kg) for one day cured infection: a single 100 mg/kg treatment removed >90% of worms. Treatment also reversed the otherwise fatal decrease in blood hemoglobin levels and body weights of hosts. Consistent with its mechanism of action, K11777 decreased by >95% the resident CP activity in parasites harvested from hamsters 8 h post-treatment with a single 100 mg/kg oral dose.

**Conclusion:**

A new, oral single-dose anthelmintic that is active in an animal model of hookworm infection and that possesses a distinct mechanism of action from current anthelmintics is discovered. The data highlight both the possibility of repurposing the anti-chagasic K11777 as a treatment for hookworm infection and the opportunity to further develop CPIs as a novel anthelmintic class to target hookworms and, possibly, other helminths.

## Author Summary

In spite of the enormous prevalence of hookworm disease, just two drugs, albendazole and mebendazole, are most commonly employed for treatment and control, and both belong to the same benzimidazole chemical class. There exists, therefore, a pressing need to develop new, safe and inexpensive agents for the treatment of human nematode infections of global significance. We report the discovery of the striking efficacy of the cysteine protease inhibitor, K11777, against hookworms both *in vitro* and *in vivo* and discuss the development of this class of compounds as novel anthelmintics for the clinical management of hookworm disease. K11777 is chemically distinct from all the current anthelmintics and, therefore, not likely to share resistance characteristics. We describe mechanism of action studies that demonstrate that cysteine protease activity in parasites recovered after *in vivo* treatment with K11777 is almost completely (>95%) abrogated. Lastly, we report that K11777 provides near cure (>90%) of hookworm infection in a single oral administration (complete cure when given twice in one day). These results suggest that K11777 is on target to meet the current clinical practice and the logistics demanded for mass drug delivery of anthelmintics to humans (i.e., oral, single-dose treatment).

## Introduction

One of a number of soil-transmitted helminthiases (STHs) that is deeply rooted in poverty, hookworm disease afflicts as much as 10% of the world's population in sub-Saharan Africa, South America, and South and South-East Asia [1], [2], [3]. The principal etiological agents in humans are the nematodes *Necator americanus* (causing necatoriasis) and *Ancylostoma duodenale* (ancylostomiasis), although *Ancylostoma ceylanicum* is found in certain locales [4], [5]. Hookworm zoonoses of minor medical importance also occur but these usually manifest with the restricted dermatitis condition of ‘cutaneous larva migrans’, (e.g., [6] and references therein).

Hookworm infection has been described as ‘silent and insidious’ [7] and to ‘drain out the vitality’ [8] of those afflicted due to chronic wasting and lethargy that has been often misconstrued as laziness [9]. Indeed, the notion of ‘draining’ is apt as most pathology arises from adult worms that attach to and feed on intestinal mucosa and blood [10]. Of the two main parasites, *A. duodenale* is the more voracious and fecund, sucking 0.1–0.2 mL blood and producing 28,000 eggs per day ([11] and references therein). The disease is most strikingly manifested in the under-nourished, not least in directly causing or exacerbating existing iron-deficient anemia that can slow physical and cognitive development in children [8], [12], [13], adversely affect fetal weight and growth, and contribute to premature birth and maternal mortality [14], [15].

Treatment and control of STHs employs periodic de-worming, particularly of school children, using a small number of well-established drugs [3], [16], [17], [18]. Of the six drugs stated in the World Health Organization's 17th Essential Medicines List for intestinal helminthiases, namely, albendazole (ABZ), mebendazole (MBZ), pyrantel pamoate, praziquantel, levamisole and niclosamide; http://www.who.int/medicines/publications/essentialmedicines/en/), the first two benzimidazoles are most commonly employed to treat hookworm infection. Of these, ABZ is the more effective as a single oral dose drug [19], [20], [21], [22]. This ease of administration, efficacy and safety record makes ABZ ideal for integration into mass drug administration (MDA) programs that aim to deliver packages of essential medicines for various tropical diseases ([17] and references therein).

Although not necessarily indicating genuine drug resistance [23], decreased efficacy against hookworm infection has been reported for MBZ [22], [24], [25] making ABZ all the more important for control of this global disease. Experience in the animal health sector indicates that resistance to one member of the benzimidazole class usually extends to other members of the same class [16], [26]. In this regard, the recent reports of less-than-expected cure rates with ABZ are disquieting [27], [28] (also a reviewed in [29] and [23]), and in one case, cannot be interpreted as either poor drug quality or a lack of patient compliance [27]. Thus, there is continued impetus not just to identify other anthelmintics (e.g., [30], [31] for two notable examples) but to eventually introduce vaccine therapy (reviewed in [32], [33]).

Here, we report the striking therapeutic effect of the Clan CA (MEROPS nomenclature [34]) cysteine protease inhibitor (CPI), K11777 (*N*-methyl-piperazine-phenylalanyl-homophenylalanyl-vinylsulfone phenyl), in a well-established animal model of hookworm infection [31], [35], [36], [37], [38] that employs the golden Syrian hamster and *A. ceylanicum*. Originating in an industrial drug development program to treat osteoporosis [39], attention to K11777's cidal activity in animal models of eukaryotic parasitism first arose with the cure of acute infection in mice by the etiological agent of Chagas' disease, *Trypanosoma cruzi* [40]. Subsequent studies have described its therapeutic benefit in various animal models for this [41] and other protozoal infections [42], [43], [44], and for the flatworm parasite, *Schistosoma mansoni* [45]. In every case, the demonstrated molecular target of anti-parasite action has been Clan CA (cathepsin) proteases. K11777 is currently progressing through Investigational New Drug (IND)-enabling studies required by the U.S. Food and Drug Administration [46] with the goal of entering clinical trials as early as 2013 for treatment of Chagas' disease. The implications of the present discovery of cure of hookworm infection in an animal model and the possible new disease indication for this drug candidate and class are discussed.

## Methods

### Parasite isolation and culture

The life cycle of *A. ceylanicum* is maintained in Golden Syrian hamsters (*Mesocricetus auratus*; Harlan Sprague Dawley, Somerville, NJ) as described [35], [36], [37]. Soluble hookworm protein extracts (HEX) were prepared [47] and protein concentrations determined using bicinchoninic acid (BCA) reagents (Pierce Biotechnology, Rockford, IL). The maintenance and care of experimental animals complied with the National Institutes of Health guidelines for the humane use of laboratory animals and were approved by the Yale University Animal Care and Use Committee.

### *In vitro* egg hatching assays (EHA)

The CPIs, K11002 and K11777, were originally part of a series of compounds provided by James Palmer of Khepri Pharmaceuticals, South San Francisco, CA [39]. Both inhibitors were in sufficient quantities to perform the comparative experiments described below. *A. ceylanicum* eggs were recovered from infected hamster feces as described [48] and placed at a density of 100 eggs/well in a 96-well plate containing ABZ (Sigma), K11002 or K11777 serially diluted in water. The total number of eggs and hatched larvae in each well were counted by light microscopy 24 h later. The total number of viable larvae, assessed based on motility, were also counted in each well. Data are expressed as the percentage of hatched larvae and the percentage of viable larvae in experimental wells relative to DMSO controls.

### *In vitro* adult worm killing assay (WKA)

To measure the effects of these CPIs on worm viability, *A*. *ceylanicum* adult hookworms were employed in a worm killing assay (WKA) as described [31], [49]. Male and female worms were recovered from the small intestines of infected hamsters, washed three times in RPMI 1640 containing 20 U/20 µg/ml penicillin/streptomycin, 10 µg/ml Fungizone (Invitrogen) and placed at a density of 10 worms/well (2 wells/treatment) into 24-well plates containing K11002 and K11777 (1–100 µM) diluted in the same medium supplemented with 50% fetal calf serum. Control wells were treated with ABZ (50 µM) or equivalent volumes of DMSO vehicle alone. To assess worm morbidity, individual worms were scored using a 5-point morbidity scale (Figure S1) at 120 h post-treatment (HPT).

### Treatment of hamsters infected with *A. ceylanicum* with K11777

Groups of hamsters (n = 6) were infected with 75 or 100 third stage *A. ceylanicum* larvae by oral gavage and followed for 24 days post-infection (DPI) to monitor blood hemoglobin levels and weight gain [37]. As indicated in the relevant figure legends, treatment regimens (commencing 17 DPI) targeted adult worms with K11777 (prepared fresh in 200 µl water, *q.d.* or *b.i.d.* for up to two days orally or 3 days intra-peritoneally (i.p.): ABZ (prepared fresh in 200 µl water) was given orally once or for 3 days. K11777 was chosen over K11002 for these experiments given its better solubility in intestinal fluids and oral bioavailability ([50], and references therein). On day 24, hamsters were sacrificed and their intestinal worm burdens counted. To measure the effect of compounds on parasite CP activity, one hamster from each of the once orally-treated K11777, ABZ and vehicle groups was sacrificed 8 h post-treatment.

### Assay for cysteine protease activity

Worms recovered from hamsters 8 h post-treatment with a single oral dose of 100 mg/kg K11777, 10 mg/kg ABZ or vehicle, were washed three times in RPMI 1640 and frozen on dry ice prior to assay. Worms were thawed in 100 µl assay buffer (0.05 M sodium acetate, pH 5.5) and homogenized using Kontes RNase-free disposable pellet pestles and microtubes for 10 min at room temperature (r.t.). Homogenates were centrifuged at 5,000 *g* for 10 min and the supernatants removed for analysis. Supernatants (1–2.5 µl) were mixed with 100 µl assay buffer containing 2 mM DTT in a black 96-well microtiter plate and left to stand at r.t. for 10 min. Then, 100 µL of assay buffer containing 2 mM DTT and 20 µM of the dipeptidyl fluorogenic substrate, benzyloxy carbonyl-phenylalanyl-arginyl-7-amido-4-methylcoumarin (Z-Phe-Arg-AMC) [51] was added with mixing. Linear rates of hydrolysis were followed in a Molecular Devices FlexStation for 10 min. In order to determine the contribution of CP activity to the total activity being measured, K11777 at 1 µM (as a 1 µl aliquot in DMSO) was added to the incubation prior to addition of substrate. Protein concentrations of supernatants were measured using the micro-Bradford assay (BioRad).

### Statistics

Worm morbidity and worm burden data were analyzed using one-way ANOVA and Tukey's Multiple Comparison Test. Hamster weight and hemoglobin data were analyzed using one-way ANOVA and Bartlett's test for equal variances. Paired t-tests were used to compare cysteine protease activities in extracts of worms recovered from hamsters. The GraphPad Prism software application (version 5.01) was employed to derive statistics.

## Results

### Cysteine protease inhibitors kill *A. ceylanicum* eggs and larvae *in vitro*

**Figure 1:**
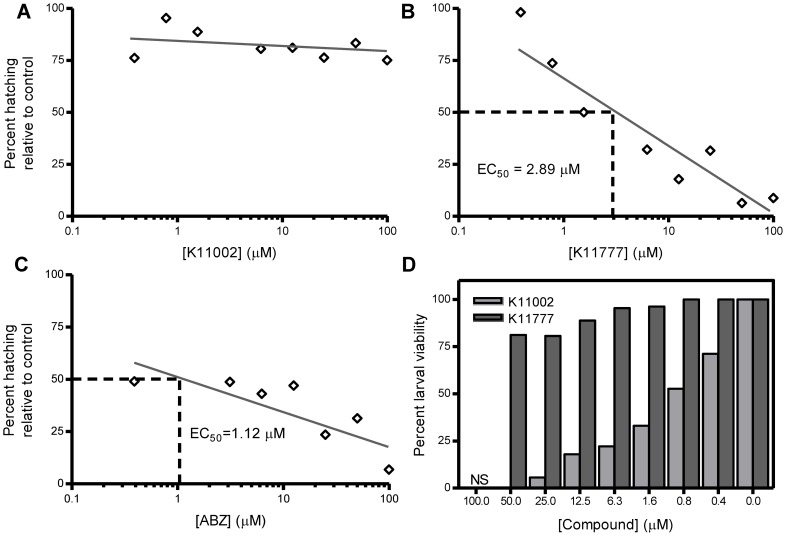
K11777 inhibits *A. ceylanicum* egg hatching whereas K11002 decreases larval viability. Eggs were recovered from hookworm-infected hamster feces and used in an egg hatch assay to measure the effect of K11002, K11777 and ABZ on egg hatching and larval viability. Data are expressed as the percentage of hatched larvae (Panels A–C) or viable larvae (Panel D) relative to controls that contained equivalent volumes of DMSO alone. NS = no survival. The results presented are representative of three separate experiments.

*A. ceylanicum e*ggs were employed in an EHA to measure the effect of K11002 and K11777 on egg hatching and larval viability (Figure 1). We observed that even at the highest concentration tested (100 µM) K11002 did not inhibit egg hatching relative to DMSO controls (Figure 1A). However, K11002 reduced larval viability in a dose dependent manner (Figure 1D, EC_50_ = 1.1 µM). After 24 h of K11002 treatment at concentrations ranging from 0.4–100 µM, newly hatched larvae were motionless and displayed wrinkled cuticles indicating death. In contrast, K11777 was a potent dose-dependent inhibitor of *A. ceylanicum* egg hatching (Figure 1B, EC_50_ = 2.89 µM) but did not impact larval viability (Figure 1D). Larvae that had hatched in wells containing K11777 were active, displaying sinusoidal movement patterns and intact cuticles. The ovicidal activity of K11777 was statistically the same as that measured for ABZ (Figure 1C, EC_50_ = 1.12 µM), which also did not interfere with larval viability (not shown).

### Cysteine protease inhibitors kill adult *A. ceylanicum in vitro*

**Figure 2:**
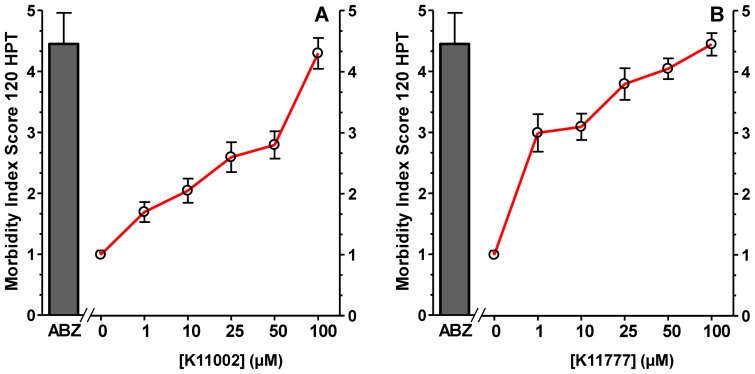
K11002 and K11777 cause morbidity in *ex vivo* cultured *A. ceylanicum* adult worms. Adult hookworms were recovered from infected hamsters and used in an adult worm killing assay to measure the effect of K11002 (Panel A) and K11777 (Panel B) on adult worm viability. Worms were scored individually using the 5 point morbidity scale (Figure S1) at 120 hours post-treatment (HPT). Control wells were treated with ABZ (50 µM) or equivalent volumes of DMSO carrier alone. Data are expressed as means ± SD of triplicate measurements. The results presented are representative of two separate experiments.

**Figure 3:**
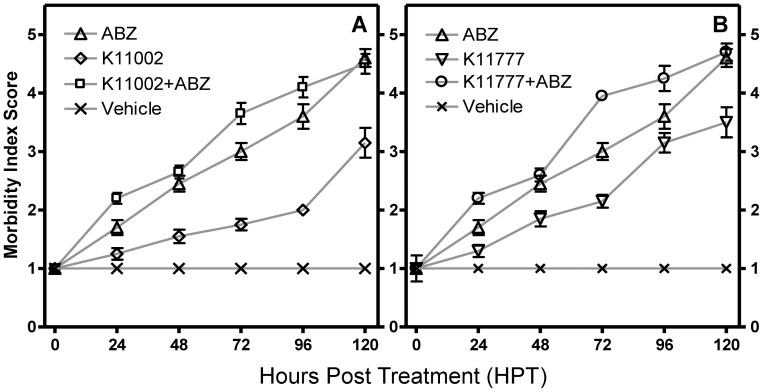
ABZ combined with K11002 or K11777 enhances morbidity in *ex vivo* cultured *A. ceylanicum* adult worms. Adult hookworms were recovered from infected hamsters and used in an adult worm killing assay to measure the effect of combining ABZ with K11002 (Panel A) or K11777 (Panel B) on adult worm survival and morbidity. Worms were scored individually using the 5 point morbidity scale (Fig S1) at different time points post-treatment. Hookworms were incubated with each compound at 25 µM. Control wells were treated with equivalent volumes of DMSO vehicle alone. Data are expressed as means ± SD of triplicate measurements. The results presented are representative of two separate experiments.

When tested in an *in vitro* WKA, K11002 and K11777 displayed different potencies in terms of inducing worm morbidity (Figure 2). For example, at 50 µM, K11002 caused significantly less morbidity (P<0.001) compared to ABZ (Figure 2A, EC_50_ = 4.5±0.51 vs. 2.8±1.01, respectively). In contrast, the morbidity caused by K11777 at 50 µM (EC_50_ = 4.05±0.76) or 25 µM (EC_50_ = 3.8±1.2) was not significantly different (P>0.05; Figure 2B) from that of ABZ at 50 µM suggesting that the CPI is as or more potent than the currently employed drug. Finally, at 72 h, combination treatment with ABZ and K11002 (each at a 25 µM) increased worm morbidity relative to treatment with either compound alone (Figure 3, P<0.01). This was also true for combining 25 µM each of ABZ and K11777 at 72 and 96 h post-treatment (P<0.001 and P<0.01, respectively).

### K11777 is a potent, single-dose oral therapy of hookworm infection in hamsters

**Figure 4:**
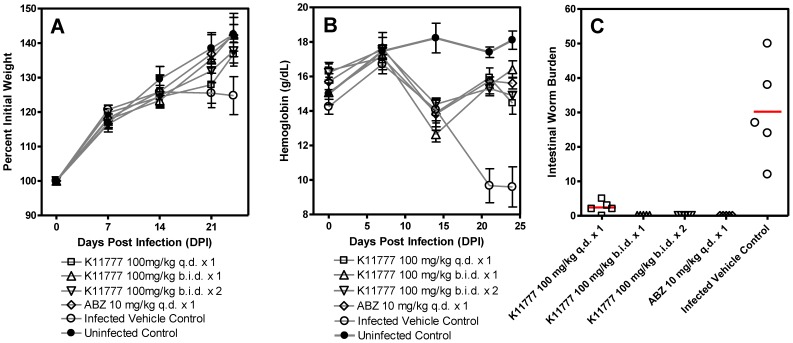
Oral administration of K11777 cures *A. ceylanicum* infection and improves blood hemoglobin levels. Groups of Syrian hamsters (n = 6) were infected with 75 third stage *A. ceylanicum* larvae and followed for 24 days to monitor blood hemoglobin levels and weight gain. At 17 days post-infection (DPI) hamsters were treated orally with K11777 (100 mg/kg *q.d.* or *b.i.d.*), ABZ (10 mg/kg *q.d*.) or vehicle alone as indicated. At 18 DPI, one group of hamsters was treated again with K11777 (100 mg/kg *b.i.d*.) and one group was treated with vehicle alone. At 24 DPI, all hamsters were sacrificed and intestinal worms counted. Compared to infected vehicle controls, hamsters treated *q.d.* or *b.i.d.* with K11777 did not show significantly improved weight gain (Panel A), but levels of blood hemoglobin were significantly higher (Panel B; P<0.001 and P<0.001 at 21 and 24 DPI, respectively). Dosing *q.d.* significantly decreased worm burdens by 90.1% (P<0.001) whereas dosing *b.i.d.* resulted in cure of infection (Panel C).

We next tested the ability of K11777 (the more soluble and bioavailable of the two CPIs *in vivo* [50]) given orally (Figure 4) or i.p. (Figure S2) to positively influence animal weight gain, blood hemoglobin levels and intestinal worm burdens as compared to infected vehicle controls. The i.p. experiments using 50 mg/kg K11777 *b.i.d.* ×3 (i.e., total daily doses of 100 mg/kg) did not significantly improve weight gain (Figure S2A), but significantly improved blood hemoglobin levels (Figure S2B; P<0.05 and P<0.001 at 17 and 24 DPI, respectively) and cured infection (Figure S2C). K11777, given orally at 100 mg/kg *b.i.d.* ×1 and ×2, did not significantly improve weight gain (Figure 4A), but significantly improved blood hemoglobin levels (P<0.001 at 21 and 24 DPI; Figure 4B) and cured infection (Figure 4C). Finally, a single oral dose of 100 mg/kg K11777 did not significantly improve weight gain (Figure 4A), but significantly improved blood hemoglobin levels (P<0.001 at 21 and 24 DPI; Figure 4B) and decreased intestinal worm burdens by 90.1% (P<0.001; Figure 4C). For comparison, a single oral dose of ABZ (10 mg/kg) did not significantly improve hemoglobin levels or hamster weights, but significantly decreased intestinal worm burdens by 100% (P<0.001, Figure 4C).

### Treatment with K11777 targets *A. ceylanicum* CP activity

**Figure 5:**
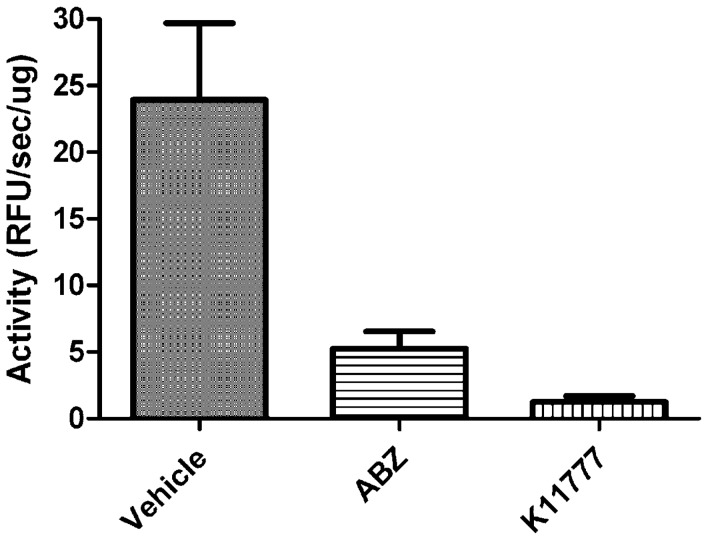
K11777 targets cysteine protease activity in *A. ceylanicum in vivo*. Soluble extracts of hookworms taken from hamsters 8 h post-treatment with single doses of ABZ (10 mg/kg), K11777 (100 mg/kg) or vehicle alone were prepared. Specific cysteine protease activity (relative fluorescence units/min/µg extract) was measured in 0.05 M sodium acetate, 2 mM DTT, pH 5.5 containing 10 µM of the dipeptidyl fluorogenic substrate Z-Phe-Arg-AMC. All activity could be inhibited by 1 µM K11777 (not shown) indicating that only cysteine protease activity was being measured. Data points are expressed as means ± SD of three separate experiments each performed in duplicate. Values for CP activity between the three experimental groups were statistically different using paired t-tests: P = 0.0024 for vehicle vs. K11777; P = 0.0074 for ABZ vs. K11777 and P = 0.0054 for vehicle vs. ABZ.

A standard assay using a dipeptidyl fluorogenic substrate for CP activity was employed to understand whether administration of K11777 8 h prior to worm recovery decreases the specific CP activity (i.e., as a function of protein concentration) relative to those activities measured post-exposure to vehicle or ABZ. Thus, soluble extracts of worms exposed to K11777 contained just 5% and 23.5% of the CP activity measured in extracts of worms exposed to vehicle (P = 0.0024) and ABZ (P = 0.0074), respectively (Figure 5). Extracts from ABZ-exposed worms contained 23% of the CP activity measured in worms exposed to vehicle (P = 0.0054). It is possible that the latter finding is due to the systemic degradation of cellular architecture and biochemistry inflicted by ABZ rather being due to direct inhibition of CP activity. In all extracts, activity could be abolished with K11777 at 1 µM (not shown) indicating that only CP activity was being measured.

## Discussion

The present data indicate that the CPIs K11777 and K11002 impair the survival of multiple life-stages of the human and animal hookworm parasite *A. ceylanicum* in a dose dependent manner *in vitro*. For K11777, EC_50_ values for killing of eggs and morbidity in adult worms were equivalent to or better than those recorded for the current therapy of hookworm disease, ABZ. In addition, for both CPIs and at some of the time-points tested, there was a significant enhancement of morbidity in the presence of ABZ over compounds used alone. Most importantly, using an animal model of hookworm infection, we demonstrate the striking therapeutic benefit of K11777 administered under different regimens. Not least, a single oral dose of 100 mg/kg removed greater than 90% of adult worms whereas *b.i.d.* treatment provided cure. Consistent with this potent worm-kill, the trajectories of blood hemoglobin levels and animal body weight that would otherwise result in death of the host were universally reversed upon therapy. Finally, worm-kill after a single oral dose of K11777 was associated with a dramatic (95%) loss of parasite CP activity, consistent with the compound's known mechanism of action (see below).

K11777 inhibits Clan CA cysteine proteases [39] including mammalian cathepsins B, L and S (reviewed in [52]), orthologs of which have been successfully targeted to effect therapy in various animal models of parasitic infection (see Introduction). Hookworms and other hematophagous nematodes devote considerable transcriptional effort into expressing a family of cathepsin B-like proteases that are associated with the parasite gut (esophagus and cecum) [53], [54], [55] and that contribute to degrading blood proteins to absorbable nutrients [56], [57], [58], [59], [60], [61]. It is likely that these proteases are targeted by K11777 as evidenced here by the almost total inhibition of CP activity in *A. ceylanicum* exposed *in vivo* to the inhibitor relative to either ABZ- or vehicle-treated controls.

The expression of hookworm proteases at the host-parasite interface, i.e., the gut, has encouraged their investigation as vaccine targets (reviewed in [57], [62]). Modest reductions in worm burdens have been recorded with hookworm cathepsins B as vaccine candidates in hamsters (*N. americanus*; up to a 29% decrease in challenge burdens [63]) and dogs (*A. caninum*; 18% [64]). Importantly, however, the latter study also showed that parasite fecundity, measured as eggs per gram of feces, was significantly decreased by 61%. In addition, the challenge worms recovered were smaller than controls and specific antibody that bound to the intestinal brush border interfered with the activity of the target cathepsin B [64]. Thus, the immunological evidence demonstration that hookworm viability can be negatively impacted through the targeting of gut cysteine proteases is powerfully underscored here with a chemical inhibitor.

Unlike the situation for most of the previous organisms tested whereby relatively long-course dosing (days or weeks depending on parasite target, e.g., [40], [45]), was needed to significantly ameliorate infection intensity and pathology, the present discovery of the hookworm parasite's sensitivity to K11777, including after a single oral treatment, is remarkable and strongly encourages further investigation for two principal reasons. First, the acute response of hookworm infection to K11777 is commensurate with the short (preferably single) oral dosing regimen currently employed for the MDA of anthelmintics [2], [17], [65]. Second, given that K11777 is already being targeted for submission as an IND to treat Chagas' disease, the inhibitor could also be tested as an experimental drug for hookworm disease. To support the potential clinical use of K11777 in treatment of hookworm infection, it will be necessary to define oral efficacy in other animal models including *N. americanus* in hamsters and *A. caninum* or *A. duodenale* in dogs.

Our results using i.p. dosing demonstrate that K11777 kills hookworm via the bloodstream. However, after oral dosing, the relative contribution of this access route and *trans*-cuticular migration to worm-killing is an open question, as it still is for some current anthelmintics [16]. Additional experimentation with a variety of CPIs that are more or less bioavailable will be required to answer this question - an answer that may influence the possible use of CPIs to treat other non-hematophagous gastro-intestinal nematodes. Pertinent to this discussion is the striking impact of CPI structure on the reciprocal activities of K11777 and (the more hydrophobic) K11002 in modulating larval viability.

Whether or not K11777 as an entity goes forward as an investigative treatment for hookworm disease, the dramatic susceptibility of the parasite *in vivo* to CP inhibition encourages a broader investigation of structural analogs and other CPI scaffolds, perhaps similar to those currently under investigation for other parasitic diseases, including malaria and Human African Trypanosomiasis [66], [67], [68]. The search might also encompass CPIs under pre-clinical and clinical investigation for treatment of non-infectious diseases such as osteoporosis and auto-immune disorders [69], [70], [71], [72] in order to take advantage of the extensive industrial experience in CPI design [71], [73].

The benzimidazoles currently used to treat hookworm disease display a spectrum of activity against other, and often co-endemic, STH's. For example, a recent meta-analysis demonstrated that the standard single 400 mg dose of ABZ, in addition to producing a cure rate (CR) of 72% against hookworms, is effective against ascariasis (CR 88%), and (markedly less so) against trichuriasis (CR 28%) [21], relative efficacies that have been confirmed more recently [74]. For CPIs, it remains to be determined whether a spectrum of activity exists, nevertheless, the discovery of this new class of hookworm anthelmintic with a distinct mechanism of action from ABZ is potentially useful given the concerns regarding the future of benzimidazoles as effective drugs. In addition, as is now common in the animal health industry [16], [75] and in the treatment of many infectious diseases of humans [76], [77], [78], drug combinations must be considered if the efficacy of the few anthelmintic drugs available is to be protected. Thus, treatment of hookworm disease and other STHs might also benefit from drug combinations in which the contribution of CPIs could be substantial by improving either anthelmintic efficacy and/or spectrum of activity. In this regard, the improved anthelmintic activity measured *in vitro* when either CPI was combined with ABZ (Figure 3) may be relevant, although further tests (e.g., employing different compound concentrations) are required to fully understand whether additive effects might be involved.

To conclude, we demonstrate a remarkable sensitivity of the hookworm parasite in an animal model of infection to the CPI, K11777. The identification of a novel chemical class of anthelmintic is encouraging given the heavy reliance on benzimidazoles to treat human hookworm disease and the threat of emerging drug resistance.

## Supporting Information

Figure S1 **Morbidity index for** ***ex vivo*** **cultured adult** ***A. ceylanicum*** **.** Hookworms are individually inspected by light microscopy at 24 h intervals out to 120 h and scored using the five point morbidity index indicated in the figure. The scoring system takes into account worm morphology and motility. Scores are expressed as means ± SD with 20 worms per compound treatment.
(TIF)

Figure S2 **Intra-peritoneal administration of K11777 cures** ***A. ceylanicum*** **infection and improves blood hemoglobin levels.** Groups of Golden Syrian hamsters (n = 5) were infected with 100 third stage *A. ceylanicum* larvae and followed for 24 days post-infection to monitor blood hemoglobin levels and weight gain. At 17–19 days post-infection (DPI), hamsters were treated with K11777 (50 mg/kg *b.i.d.*) dissolved in deionized water (200 µL per administration), ABZ (10 mg/kg *q.d.* ×3) dissolved in deionized water (200 µL per administration) or vehicle alone. At 24 DPI, all hamsters were sacrificed and intestinal worms counted. Compared to infected vehicle controls, treatment with K11777 did not significantly improve weight gain (Panel A), but significantly improved blood hemoglobin levels (Panel B; P<0.001 and P<0.05 days 17 and 24 DPI, respectively) and provided cure (Panel C; P<0.001).
(TIFF)
