# Supplementary figures and images for: Cure of Hookworm Infection with a Cysteine Protease Inhibitor
Source: PLoS Negl Trop Dis. 2012 Jul 3;6(7):e1680. doi: 10.1371/journal.pntd.0001680 (PMC3389033; doi:10.1371/journal.pntd.0001680)

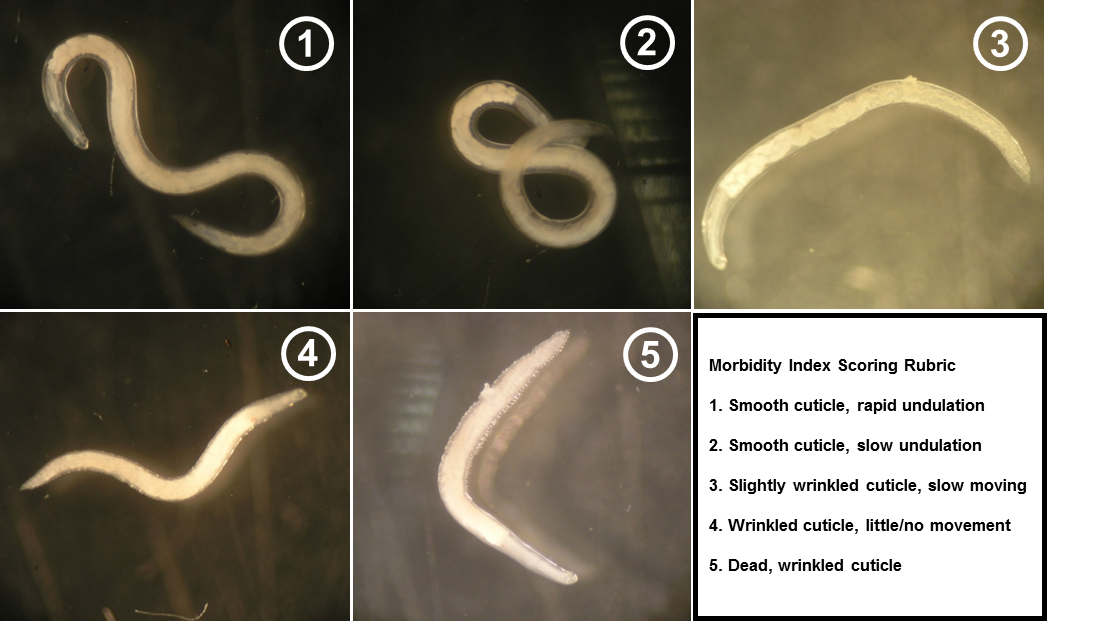

Supplement: Figure S1 — Morbidity index for ex vivo cultured adult A. ceylanicum . Hookworms are individually inspected by light microscopy at 24 h intervals out to 120 h and scored using the five point morbidity index indicated in the figure. The scoring system takes into account worm morphology and motility. Scores are expressed as means ± SD with 20 worms per compound treatment. (TIF) [file pntd.0001680.s001.tif]

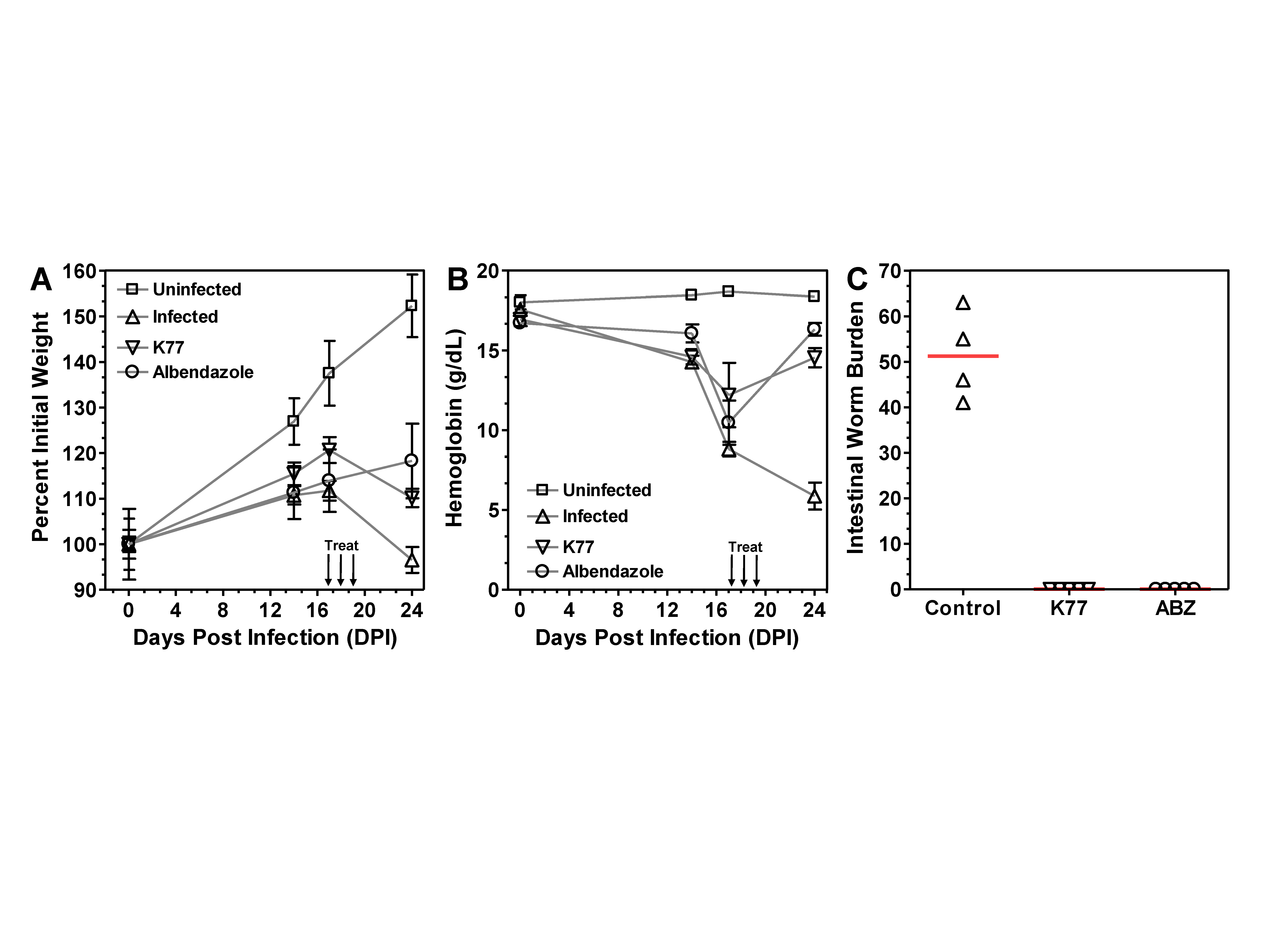

Supplement: Figure S2 — Intra-peritoneal administration of K11777 cures A. ceylanicum infection and improves blood hemoglobin levels. Groups of Golden Syrian hamsters (n = 5) were infected with 100 third stage A. ceylanicum larvae and followed for 24 days post-infection to monitor blood hemoglobin levels and weight gain. At 17–19 days post-infection (DPI), hamsters were treated with K11777 (50 mg/kg b.i.d.) dissolved in deionized water (200 µL per administration), ABZ (10 mg/kg q.d. ×3) dissolved in deionized water (200 µL per administration) or vehicle alone. At 24 DPI, all hamsters were sacrificed and intestinal worms counted. Compared to infected vehicle controls, treatment with K11777 did not significantly improve weight gain (Panel A), but significantly improved blood hemoglobin levels (Panel B; P<0.001 and P<0.05 days 17 and 24 DPI, respectively) and provided cure (Panel C; P<0.001). (TIFF) [file pntd.0001680.s002.tif]
